# Dyslipidemia in Transplant Patients: Which Therapy?

**DOI:** 10.3390/jcm11144080

**Published:** 2022-07-14

**Authors:** Gabriella Iannuzzo, Gianluigi Cuomo, Anna Di Lorenzo, Maria Tripaldella, Vania Mallardo, Paola Iaccarino Idelson, Caterina Sagnelli, Antonello Sica, Massimiliano Creta, Javier Baltar, Felice Crocetto, Alessandro Bresciani, Marco Gentile, Armando Calogero, Francesco Giallauria

**Affiliations:** 1 Department of Clinical Medicine and Surgery, University of Naples Federico II, Naples, Via S. Pansini 5, 80131 Naples, Italy; gabriella.iannuzzo@unina.it (G.I.); mariatripaldella@gmail.com (M.T.); vaniamall2017@gmail.com (V.M.); paola.iaccarinoidelson@gmail.com (P.I.I.); margenti@unina.it (M.G.); 2 Department of Translational Medical Sciences, University of Naples Federico II, Naples, Via S. Pansini 5, 80131 Naples, Italy; gianluigi.cuomo95@gmail.com (G.C.); dilorenzoanna2@gmail.com (A.D.L.); francesco.giallauria@unina.it (F.G.); 3 Department of Mental Health and Public Medicine, University of Campania “Luigi Vanvitelli”, Via S. Pansini 5, 80131 Naples, Italy; caterina.sagnelli@unicampania.it; 4 Department of Precision Medicine University of Campania “Luigi Vanvitelli”, Naples, Via S. Pansini 5, 80131 Naples, Italy; antonello.sica@fastwebnet.it; 5 Department of Neurosciences, Reproductive Sciences and Odontostomatology, University of Naples Federico II, Naples, Via S. Pansini 5, 80131 Naples, Italy; massimiliano.creta@unina.it (M.C.); felice.crocetto@unina.it (F.C.); 6 Servicio de Cirugía General, Xerencia de Xestión Integrada de Santiago (XXIS/SERGAS), 15706 Santiago de Compostela, Spain; javier.baltar.boileve@sergas.es; 7 Department of Medicine and Medical Specialties, A. Cardarelli Hospital, 80131 Naples, Italy; alessandro.bresciani@aocardarelli.it

**Keywords:** cardiovascular disease, dyslipidemia, immunosuppressive therapy, organ transplant

## Abstract

Cardiovascular disease is the most important cause of death worldwide in recent years; an increasing trend is also shown in organ transplant patients subjected to immunosuppressive therapies, in which cardiovascular diseases represent one of the most frequent causes of long-term mortality. This is also linked to immunosuppressant-induced dyslipidemia, which occurs in 27 to 71% of organ transplant recipients. The aim of this review is to clarify the pathophysiological mechanisms underlying dyslipidemia in patients treated with immunosuppressants to identify immunosuppressive therapies which do not cause dyslipidemia or therapeutic pathways effective in reducing hypercholesterolemia, hypertriglyceridemia, or both, without further adverse events.

## 1. Introduction

Atherosclerosis is defined as the accumulation of fatty and fibrous material in the intima layer of an artery, inducing formation of atheroma (i.e., atherosclerotic plaque). Over time, plaque continues to grow, increasing its calcium and fibrous material content, and it can lead to tissue ischemia by obstructing the lumen of the vessel or disrupting itself by occluding the lumen of a distal vessel [1]. Depending on the affected artery, atherosclerotic cardiovascular disease (ASCVD) can cause acute coronary syndromes (ACS), ischemic stroke or transient cerebral ischemic attacks (TIA), and peripheral artery disease (PAD). 

In 2019, there were an estimated 523 million cases of cardiovascular diseases (CVD), causing 18.5 million deaths [2]. Moreover, CVDs are a relevant cause of disability, bringing about 194 million and 143 million disability-adjusted life years (DALYs) for ischemic heart disease and stroke, respectively [2].

Dyslipidemia, that is, an alteration in lipid metabolism, is a well-known risk factor for ASCVD development; in particular, low-density lipoprotein cholesterol (LDL) circulating levels are unequivocally established as the principal determinant of atherosclerotic plaque formation and progression [3]. 

Atherosclerosis is a long process, beginning in the first decades of life, even in people without specific genetic characteristics, regardless of the presence of symptoms [4]. 

In addition to lipids, other risk factors have been identified in atherosclerosis, such as hyperglycemia, hypertension, tobacco use, and visceral adiposity [5,6,7,8].

Moreover, chronic kidney disease (CKD) is a well-known independent risk factor for atherosclerosis, and hemodialysis patients have a higher inflammatory status and more severe impaired blood flow [9].

All the above-mentioned factors contribute to plaque formation, triggering activation of inflammatory pathways, with consequent accumulation of fibrous material and plaque growth [1].

In patients who underwent solid organ transplantation and who were subjected to immunosuppressive therapies, dyslipidemia is very common; consequently, CVDs represent a frequent cause of long-term mortality in these patients, being estimated as the first cause of death in heart and kidney transplant recipients [10,11], and the second in liver transplant recipients [12].

Particularly in heart transplant recipients, atherosclerosis seems to be more aggressive, increasing the risk of vasculopathy progressively every 5 years, with an additional risk of 10% every 2 years after the transplant [13]. In addition, in these patients a particular form of coronary atherosclerosis was found, named cardiac allograft vasculopathy (CAV), which is morphologically different from typical atheromatous plaque [14]. 

Several risk factors are involved in the pathogenesis of the atherosclerotic process in these patients, such as transplant rejection, hypertension, dyslipidemia, and diabetes; however, hypercholesterolemia and hypertriglyceridemia are the most frequent metabolism abnormalities in clinical practice [15]. Immunosuppressor-mediated hyperlipidemia is characterized by an increase in LDL cholesterol, VLDL cholesterol, and/or an increase in total plasma triglycerides, mainly VLDL triglycerides [16,17].

The aim of this review is to clarify the pathophysiological mechanisms underlying dyslipidemia in patients treated with immunosuppressants, presenting the effects of these drugs on metabolism and the atherosclerotic process. 

## 2. Role of Dyslipidemia in Atherosclerosis

Dyslipidemia is defined as an abnormal concentration of lipids in the blood, and it can be present despite normal total cholesterol levels if there is an increase of lipoproteins that carry the cardiovascular risk factor.

Lipoproteins are constituted of lipids (such as cholesterol and triglycerides) and proteins called apolipoproteins. Different lipoproteins are distinguished by size, lipid content, and type of apolipoprotein. 

Low-density lipoproteins (LDL) are small molecules, rich in ApoB-100 apolipoprotein, and are unequivocally correlated with ASCVD [3].

Very low-density lipoproteins (VLDL) and their remnants are rich in triglycerides and are also associated with ASCVD; however, this association seems to be related to the blood concentration of ApoB-containing particles rather than the concentration of triglycerides itself [18].

High-density lipoproteins (HDL) are the smallest lipoproteins and are abundant in apolipoprotein ApoA-I and ApoA-II; their function is to pick up cholesterol, which is internalized and carried to the liver or steroidogenic organs. Probably due to this role, HDL circulating levels are inversely associated with ASCVD [19], but there is no evidence that increasing these levels could reduce cardiovascular risk [20]. 

Lipoprotein(a) (Lp(a)) is similar to LDL but contains apolipoprotein Apo(a) in addition to ApoB. This particle, thanks to its small diameter, can pass through the endothelial barrier, provoking atherogenesis. Moreover, because they have a structure similar to plasminogen, pro-coagulant and pro-inflammatory effects have been shown [21]. 

The association between higher Lp(a) circulating concentrations and increased CVD risk has been assessed [22,23]; furthermore, a reduction in these levels in patients treated with proprotein convertase subtilisin/kexin 9 (PCSK9) inhibitors was shown to reduce CV risk [24,25].

Atherosclerosis is a long-lasting process, but dyslipidemia has been shown to play a key role at different stages. 

In the initiation phase, the LDL particles, with a high cholesterol content, accumulate in the innermost layer (intima) of the vessel [26]. Here, these particles undergo an oxidation reaction and are phagocytosed by macrophages, which are transformed into foam cells [26]. As result, a localized inflammatory reaction begins, with the consequent release of cytokines and expression of adhesion molecules, attracting other monocytes circulating in the blood, transforming them into macrophages which in turn become foam cells [26]. 

Since inflammation plays a pivotal role in the atherosclerotic process, C-reactive protein (CRP) has been chosen as a prognostic marker for cardiovascular risk [27]. Of note, type 1 T helper lymphocytes (producing cytokines such as IFN-γ and TNF) have been found in human atherosclerotic plaques as promotors of atherogenesis, while regulatory T cells seem to mitigate this process [28,29].

In physiological conditions, the arterial endothelium has intrinsic properties preventing thrombus formation; however, the alteration of endothelium function, which loses its permeability and nitric oxide (NO)-mediated vasorelaxation capacity [30,31], may contribute to thrombus formation. In addition, other factors, such as flow alterations, may influence atherosclerosis initiation; indeed, plaques tend to form at flow disturbance sites, such as at the branching of vessels [32]. 

During plaque enlargement, due to high lipid levels, calcification of plaque may occur [33]; if calcification is very extended, plaque disruption and consequent thromboembolic events will be less probable [34]. 

Finally, plaque evolution and consequent complications depend on fibrous cap thickness and lipid core quantity. A thin fibrous cap determines more vulnerability and probable rupture [35]; when rupture of an atherosclerotic plaque occurs, the consequent exposure of thrombogenic material that is in the core (such as tissue factor) and circulating (thrombin) triggers formation of a thrombus [35]. 

Otherwise, when plaque has lower inflammatory cells and lipid content, greater collagen matrix content and thicker fibrous cap, a different complication called “plaque erosion” may occur, leading to formation of platelet-rich “white” clots. 

The complication discussed so far typically occurs in coronary arteries, causing myocardial infarction [36]. In other vessels, atherosclerotic plaque growth may continue uninterrupted until the formation of flow-limiting lesions, causing PAD.

In addition to dyslipidemia, other risk factors contribute to the atherosclerotic process. Arterial hypertension causes endothelial dysfunction through shear stress [32], and in addition it increases levels of angiotensin II, which activates the prescription of nuclear factor-κB (NF-κB), responsible for inflammatory pathways [37]. 

Type 2 diabetes mellitus causes insulin resistance and accumulation of visceral adipose tissue, which contains inflammatory cells and increases circulating pro-inflammatory cytokines levels [5].

In conclusion, several factors are demonstrated favoring plaque formation, but dyslipidemia is certainly the main atherogenesis promoter, on which an intervention is desirable.

## 3. Dyslipidemia and Atherosclerosis in Transplant Recipients

In recent years, advances made in the field of solid organ transplants, as regards surgical techniques, infection prevention, and immunosuppressive therapy, have resulted in increasing survival of transplant recipients. Consequently, recent data have shown an increasing prevalence of CVD, which has become the leading cause of death following kidney [10] and heart transplant [11], and the second leading cause after liver transplant [12,38,39]. It should be considered that kidney transplantation can be a confounding factor, as it is often the final stage of long-lasting CKD, which is itself an important cardiovascular risk factor [40], although this had been underestimated by cardiovascular risk prediction models such as the Framingham Risk Score [41,42]. The latest European Society of Cardiology (ESC) guidelines on cardiovascular disease prevention have defined patients with moderate CKD as high risk for CVD, and those with severe CKD at very high risk [43].

For other solid organ transplant recipients, among causes of this increase in CVD, it should be considered that dyslipidemia occurs frequently in these patients.

The pathophysiological mechanisms through which an increase in circulating LDL levels favors atherosclerosis and consequently ASCVD have been presented in the previous chapter.

Prevalence of hyperlipidemia was estimated at 80% in kidney transplant recipients [44], 50% in heart transplant recipients [45], and about 70% in liver transplant recipients [15], compared to about 35% in the general population [46,47,48,49,50].

Multiple factors contribute to lipid alterations, such as genetic predisposition [44,45,46], dietary habits [51], and age; however, the main effect is due to immunosuppressants, which have shown intrinsic pharmacodynamic properties to cause dyslipidemia and hyperglycemia [52,53]. In line with these findings, serum total cholesterol concentration is higher in the first 3–6 months after transplantation, when immunosuppressants are administered at higher doses [54]. 

These drugs are responsible not only for the increase in LDL and triglycerides but are also associated with other atherosclerosis promoter factors. Arterial hypertension is a common side effect of immunosuppressants such as cyclosporine [55] and tacrolimus [56] and has been found in approximately 80% of transplant recipients [57]; corticosteroids, the oldest and most used immunosuppressants, have a well-known ability to cause hyperglycemia and diabetes [58], and this effect has been shown to be enhanced by cyclosporine and tacrolimus [59].

Furthermore, dyslipidemia can cause other complications, in addition to CVD, in solid organ transplant recipients. In kidney transplant recipients there is a higher concentration of oxidized LDL (oxLDL) particles [60], possibly due to increased inflammatory state [61] or higher concentration of pro-inflammatory triglycerides [62]. 

These higher oxLDL, levels, together with the proven lower HDL concentration, are associated with chronic allograft nephropathy (CAN) [63], which is the main cause of kidney transplant failure [64,65].

Finally, among the factors promoting atheroma formation and growth, a protracted activation of inflammation in transplant recipients has been demonstrated [66,67]. A particular consequence of inflammation, the cardiac allograft vasculopathy (CAV), has been observed after heart transplantation [68].

CAV is a particular form of coronary disease, which is responsible for about 10% of deaths after heart transplant [68]; it is distinguished from normal coronary atherosclerosis because these lesions, which appear as concentric intimal hyperplasia obliterating the lumen of the vessel, affect the intramuscular arteries and the microvascular bed [69]. 

The pathogenesis is mainly due to the formation of antibodies against donor antigens, which trigger an inflammatory response mediated by T lymphocytes directed against donor endothelial cells, which consequently proliferate and occlude the vessel lumen [70,71]. However, in addition to inflammation, other metabolic factors, including dyslipidemia, are also promoters of CAV [71].

**Figure 1:**
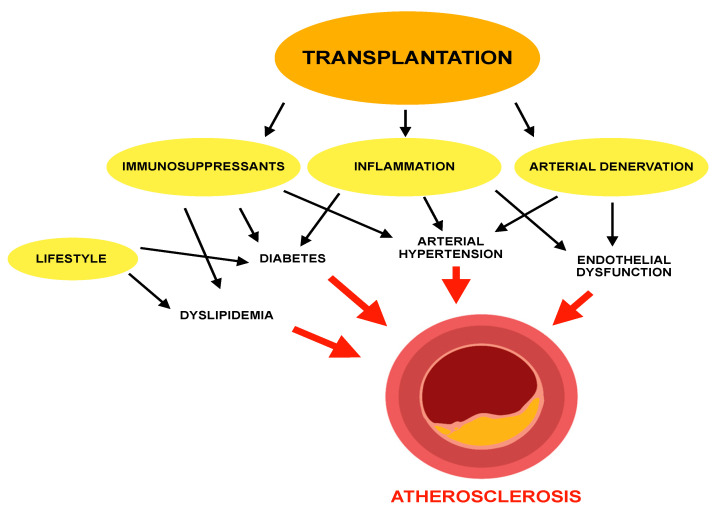
Mechanisms of atherosclerosis in transplant patients.

Mechanisms of atherosclerosis in transplant patients are illustrated in Figure 1.

In addition to the effects on cardiovascular risk, increased circulating lipid levels can cause other diseases; in fact, it has been shown that when they are in excessive levels, lipids can also accumulate in non-adipose tissue and cause lipotoxicity, which mainly affects cardiac, skeletal muscle, and kidney tissues [72]. Furthermore, it must be emphasized that lipids have a strong pro-inflammatory effect, being able to enhance the release of molecules such as adipokines [73]. 

Therefore, atherogenesis and consequently ASCVD in transplant recipients can be considered the consequence on the one hand of the inflammatory state due to immune response against donor cells and on the other hand of the drugs administered to reduce this inflammatory response and transplant rejection. 

## 4. Immunosuppressants Effect on Dyslipidemia and Other CVD Risk Factors

Immunosuppressants act on the immune cascade, depressing or suppressing the immune response at different levels, inducing a general state of immunosuppression. 

These drugs have significantly improved survival in transplant patients [74] and are, therefore, fundamental in maintenance therapy; unfortunately, they are associated with several alteration in metabolism and increasing incidence of CVD [52]. 

Depending on pharmacodynamic action, immunosuppressants are divided into different classes; in this chapter the effects of various drugs on metabolism will be analyzed.

Glucocorticoids are the oldest class of immunosuppressants. Glucocorticoids currently used in clinical practice are synthetic, such as prednisone, beclomethasone, and fluticasone. They have minimal mineralocorticoid activity, thus reducing the effects on mineral imbalance and fluid retention [75]. Glucocorticoids have potent anti-inflammatory effects, mainly by crossing the cell membrane and regulating gene transcription [76,77]. 

On the other hand, these drugs induce insulin resistance, favoring hyperglycemia and lipolysis [78], and enhance free fatty acid (FFA) synthetase and acetyl-coenzyme carboxylase function [79], consequently increasing circulating FFAs, which are taken by the liver and become substrates for VLDL. 

Moreover, glucocorticoids act on lipoproteins by various mechanisms: they increase VLDL conversion into LDL by reducing lipoprotein lipase (LPL) activity, which favors the clearance of chylomicrons and VLDL [80]; they increase the activity of hydroxy-methylglutaryl coenzyme A (HMG-CoA) reductase, which has a main role in cholesterol synthesis [81]; they downregulate expression of LDL receptors, reducing the removal of these particles from circulation [81]. The result of these mechanisms is an increase in triglycerides and total cholesterol, estimated up to 50% in patients on immunosuppressive therapy [82].

In addition, glucocorticoids increase the risk for CVD provoking arterial hypertension [83] and post-transplantation diabetes mellitus (PTDM) or temporary diabetes [58]. Fortunately, the early steroid withdrawal strategy (3 months after transplant) has been shown to be effective in reducing hyperlipidemia, hypertension, and diabetes, without adverse effects on renal function and patient survival at 3 years [84].

Calcineurin inhibitors act on the phosphatase activity of calcineurin and consequently dephosphorylation of the nuclear factor of activated T cells (NFAT), which when translocated into the nucleus promotes interleukin-2 (IL-2) and IFN-γ transcription [85]. The most used drugs of this class are cyclosporine (CsA) and Tacrolimus.

CsA enhances the activity of hepatic lipase and reduces the activity of LPL, thus increasing the concentration of FFA [86] and, therefore, as mentioned above, also of VLDL and LDL. Furthermore, it can cause insulin resistance by inhibiting the secretion of pancreatic β-cells [78], resulting in lipid changes similar to those caused by glucocorticoids. 

Moreover, CsA acts on cholesterol levels through other modalities: it inhibits cholesterol transformation into bile acid, so reducing its export from the liver [87]; it has been hypothesized that CsA is internalized through LDL receptors [88,89], and this may reduce the clearance of circulating LDL; furthermore, CsA concentrations have been shown to be associated with an increase in oxidation of LDL particles [90], which appear to have a greater ability to cause CVD [3]. 

A significant increase of 21% in total cholesterol and 31% in LDL in patients treated exclusively with CsA has been reported [83]. CsA appears to increase total cholesterol levels less markedly than glucocorticoids (18% vs. 27%) [91], while a more marked increase in triglycerides has been found (25% vs. 12% with prednisone) [82]. 

However, combination therapy of CsA and glucocorticoids greatly increases LDL and triglycerides levels [82,91].

Tacrolimus has a mechanism of action and effects on lipid and metabolism similar to CsA but has shown a significantly reduced risk of graft failure [92], a lower increase in LDL levels, and lower PTDM incidence [93]. However, a recent meta-analysis by Kotha et al. [94] seems to overturn this evidence, suggesting that the risk of developing PTDM is higher with tacrolimus than with CsA (OR = 1.4; 95% CI: 1.0–2.0). On the other hand, a randomized controlled trial by Torres et al. [95] compared different tacrolimus–steroid and cyclosporine–steroid combination regimens, concluding that, although the incidence of PTDM was higher in patients treated with regimens involving tacrolimus than in cyclosporine, the best balance between PTDM and acute rejection incidence is reached in a tacrolimus-based immunosuppression regimen.

However, both calcineurin inhibitors have been shown to increase arterial hypertension early after starting [96,97], due to alterations in vascular reactivity, regulation of intracellular calcium, and enhancing production of vasoconstrictors [55,98].

Inhibitors of the mammalian target of rapamycin (mTOR), sirolimus, and everolimus, are strongly associated with an alteration in lipid metabolism, due to their mechanism of action; in fact, mTOR complex-1 [99] is involved in lipoprotein synthesis and insulin resistance. Thus, these drugs inhibit LPL function, reduce the catabolism of apolipoproteins apoB100 and apoCIII, alter insulin secretion and induce pancreatic β-cells apoptosis [78], resulting in an increase in triglycerides, VLDL, and LDL [100]. It has been demonstrated that patients treated with sirolimus had higher levels of total cholesterol and triglycerides compared to other immunosuppressants [53]. 

In addition, everolimus showed a marked increase in total cholesterol (47.4 mg/dL, 95% CI 37.5–57.3) and triglycerides (28.9 mg/dL, 95% CI: 20.7–37.1) in a 2015 meta-analysis versus placebo [101]. On the other hand, mTOR inhibitors showed similar alterations on hyperglycemia and weight gain compared to other immunosuppressants, but a lower effect on arterial hypertension [53]. However, a systematic review by Karpe et al. [102] showed that calcineurin inhibitor substitution with mTOR inhibitors increased the risk of graft rejection, and this can affect the choice of the immunosuppressant.

Antiproliferative agents Azathioprine (AZA) and Mycophenolate Mofetil (MMF) are purine analogues used in organ transplant as glucocorticoid-sparing agents. There is no evidence that these drugs could elevate lipid levels or have other effects on metabolism [52]. The replacement of glucocorticoids by AZA in renal transplant recipients, although interrupted in some patients due to hematological adverse effects, has shown a reduction in lipid levels and a lower requirement for antihypertensive drugs use [103]. In liver transplant recipients, the immunosuppressive MMF-containing regimen showed a lower risk of CV mortality [104]. 

Currently, there is little evidence about the role of monoclonal and polyclonal antibodies. Therefore, it is difficult to address this topic in the present review. Although these drugs currently have a greater risk for graft failure compared to previously cited immunosuppressants [105], they have shown a lower incidence of cardiovascular effects [106] and may also prove effective in the future as rescue therapies for immunosuppressants-induced nephrotoxicity [107] or late allograft rejection [108].

Immunosuppressants effects on lipids and other CV risk factors are summarized in Table 1.

## 5. Management of Dyslipidemia in Transplant Recipients

Due to the well-known increase in CVD in transplant recipients, the latest ESC guidelines on dyslipidemia consider these patients as a category in need of special attention [109]. However, these patients are not assigned to a risk category for the transplant itself, but for any other known risk factors. For example, transplant recipients who have experienced previous ACS or stroke or are affected by diabetes mellitus with organ damage are considered at very high risk for CVD, and, therefore, the treatment of dyslipidemia aims to achieve LDL levels < 55 mg/dL. Kidney transplant recipients are an exception because they are all considered at very high risk as end-stage CKD belongs to this category [109,110].

Therefore, for other transplant patients there is no specific risk class or LDL target to be achieved. Nevertheless, taking into account the high lipid levels that can be reached due to immunosuppressants, it should be stated that patients with total cholesterol levels > 310 mg/dL or LDL > 190 mg/dL are to be considered at high risk for CVD, therefore, adopting a therapeutic regimen that achieves LDL levels < 70 mg/dL is indicated [109].

Furthermore, treating dyslipidemia in transplant recipients reduces not only the incidence of CVD, but also other complications related to increased lipid levels such as CAN [65]; in addition, in heart transplant recipients, keeping LDL levels < 100 mg/dL has been shown to reduce the risk of developing CAV [111].

Different options are applicable in transplant recipients, depending on the CV risk and the transplant rejection risk.

First, lifestyle modifications should be encouraged, because drug-induced dyslipidemia is amplified by a sedentary lifestyle with high intake of saturated fat, reduced physical activity and obesity, also related to the stimulation of appetite induced by cortisones. The Mediterranean diet has been shown to reduce total cholesterol and LDL levels by about 10% in renal transplant recipients, and a statistically significant reduction in triglycerides in patients with lower LDL levels [112]. 

Before starting pharmacological therapy, special consideration must be given to pharmacological interactions, which are ultimately the determining factor in therapy choice [113].

Therefore, it is possible to initially consider reducing the dosage of immunosuppressants or replacing drugs with greater hyperlipidemic effect, such as CsA and mTOR inhibitors. Steroid withdrawal showed a significant reduction in total cholesterol, LDL, and triglycerides, but also in HDL [103,114]; use of deflazacort, a particular steroid compound with less effect on insulin resistance, could improve metabolic parameters in transplant recipients [115]; furthermore, conversion from CsA or sirolimus to tacrolimus resulted in better control of lipid levels [53,116,117,118,119], even in patients already treated with lipid-lowering agents ^110^. The use of a low-CsA or standard-CsA regimen did not lead to significant differences in cholesterol levels [53].

Statins are indicated by ESC guidelines as first-line drugs for dyslipidemia in transplant recipients (recommendation level II A) [109]. These drugs showed a significant reduction in total cholesterol (mean reduction −42.43 mg/dL: CI −51.22 to −33.64), LDL (mean reduction −43.19mg/dL: CI −52.59 to −33.78), and triglycerides (mean reduction −27.28 mg/dL: CI −34.29 to −20.27) [120]. In addition, reduction in major CV events (RR 0.84: CI 0.66 to 1.06), CV mortality (RR 0.68: CI 0.45 to 1.01), and myocardial infarction (RR 0.70: CI 0.48 to 1.01) were observed [120]. Furthermore, to reduce lipid levels, statins were found to be very useful for their pleiotropic effects, such as improvement of endothelial function and the ability to modulate inflammation, which is responsible not only for CVD but also for transplant rejection [121,122,123,124].

However, statins should be used with particular care in these patients, due to potential drug interaction. In fact, calcineurin inhibitors, mainly CsA, and mTOR inhibitors are both metabolized by cytochrome CYP3A4, which is involved in atorvastatin, lovastatin, and simvastatin metabolism [113]. In addition, CsA acts as both an inhibitor and a substrate for permeability glycoprotein (P-gp) [125], which has as substrates atorvastatin, lovastatin, and pravastatin; for all the reasons mentioned above, concentrations of both immunosuppressants and statins (and consequently the risk of serious side effects such as statin rhabdomyolysis) may increase [126]. For this reason, statins that are metabolized from other cytochromes, such as CYP2C9 for rosuvastatin and fluvastatin, or minimally involved in cytochrome metabolism, such as pravastatin, should preferably be used [113,127].

However, using statins at lower dosage in combination with immunosuppressants which showed less adverse effects, such as tacrolimus, sirolimus, or everolimus, is an acceptable strategy [13,113,128].

Ezetimibe is considered a second-line therapy in combination with statins to reach lower cholesterol levels, or in monotherapy for those patients who have experienced adverse events or intolerance (ESC guidelines recommendation level II B) [109]. 

Several trials have assessed ezetimibe efficacy in statistically significant reduction of total cholesterol, LDL, and triglycerides in different solid organ transplantations [129,130]. 

Elevation in both ezetimibe and CsA serum levels have been found in the co-administration strategy, so a lower dose of ezetimibe (5 mg) should be considered in these patients [13].

Fibrates, in particular gemfibrozil and fenofibrate, have been shown to be effective in reducing triglyceride and VLDL levels and increasing HDL levels in solid organ transplant recipients [131,132,133]. However, fibrates are CYP3A4 inductors, causing a decrease in CsA and everolimus plasma concentrations, therefore a higher frequency of rejection has been noted [134,135]. Moreover, fibrates and statins co-administration presents a high risk of myotoxicity, and the addition of CsA increases this risk [113].

Niacin, a vitamin B3 compound, has been shown in the general population to reduce triglyceride and LDL concentrations and incidence of CVD, in monotherapy and in addition to statins [136,137]; however, there is limited but encouraging evidence of their use in transplant recipients [138]. However, niacin should be used carefully in these patients as it can cause myopathies, gastrointestinal intolerance, hepatotoxicity, and exacerbate the side effects of immunosuppressants [13,139]. 

Bile acid sequestrants, such as cholestyramine and colesevelam, are resins which bind bile in the gastrointestinal tract, disrupting reabsorption of bile acids and increasing liver utilization of cholesterol. Due to their effect on the gastrointestinal system, bile acids can bind and thus reduce absorption of MMF, tacrolimus, and CsA, and plausibly also of mTOR inhibitors and steroids, thus decreasing their plasma levels [13,113]; however, by distancing the intake of bile acid sequestrants and immunosuppressants by at least 4 h, it is possible to reduce the interactions [140].

The omega-3 fatty acids Eicosapentaenoic acid (EPA) and Docosahexaenoic acid (DHA), in high doses of up to 6 g per day, have proven to be effective in reducing CVD and lipid levels (particularly hypertriglyceridemia and VLDL) [141] also in transplant recipients [142], without significant side effects, except for a minimal increased risk for bleeding and hypertransaminasemia, which is also a common side effect for some immunosuppressants. Moreover, positive effects on cardiac hemodynamics, renal function, inflammation, and graft survival have been shown [143,144]. Minimal drug interaction with CsA has been described, due to CYP3A4 metabolism, with possible increase in CsA serum concentration, but no differences in rejection have been found, thus their use is recommended for the treatment of hypertriglyceridemia, with careful monitoring [113].

The proprotein convertase subtilisin/kexin 9 (PCSK9) inhibitors alirocumab and evolocumab are human monoclonal antibodies directed against PCSK9, which is a protein that reduces the expression of LDL receptors and consequently LDL clearance from plasma. These drugs have been assessed to be very effective in the general population in reducing LDL, triglycerides and the more atherogenic Lp(a), with a resulting reduction in CV events [145,146]. 

To date, there is little evidence in transplant recipients [147], but PCSK9 inhibitors have been shown to be a very effective treatment for dyslipidemia, with a reported reduction in LDL values of up to 50% [148]. Moreover, PCSK9 seems to be safe in these patients because they are not involved in CYP3A4 or P-gp metabolism; however, reductions in CsA and sirolimus levels have been reported, without negative effects on rejection [148].

New lipid-lowering drugs have been recently put on the market, but their use is still too limited to have evidence in transplant patients; however, it is hoped that in future these may be a further option in patients with difficult to manage dyslipidemia.

Mipomersen is an antisense oligonucleotide which leads to degradation of ApoB-100 mRNA and consequently reduction in LDL and Lp(a) [149]. This drug use is evaluated only in FH patients, so only a few cases are reported in patients with FH who undergo heart transplantation [13]. Mipomersen seems to have no direct drug interaction, but it could aggravate liver toxicity due to some immunosuppressant drugs [13].

Lomitapide interferes with VLDL formation and is effective in LDL reduction in addition to statins in homozygous patients with FH. However, lomitapide inhibits P-gp and is involved in CYP3A4 metabolism, so potentially it can increase the serum levels of immunosuppressants, particularly calcineurin and mTOR inhibitors [13].

Finally, bempedoic acid is a novel molecule which inhibits ATP citrate lyase, an enzyme involved in cholesterol genesis. Although they act on the same pathway, it can be used in addition to statins. Since it does not interact with CYP3A4, in the future bempedoic acid could become the drug of choice in patients who must use CsA or in whom statins are not usable due to drug interaction. 

The choice of the best lipid-lowering therapy in transplant recipients must, therefore, be very careful, and several factors should be taken into consideration. 

In Table 2 lipid-lowering drugs benefits and their possible interaction with immunosuppressants are schematized.

**Figure 2:**
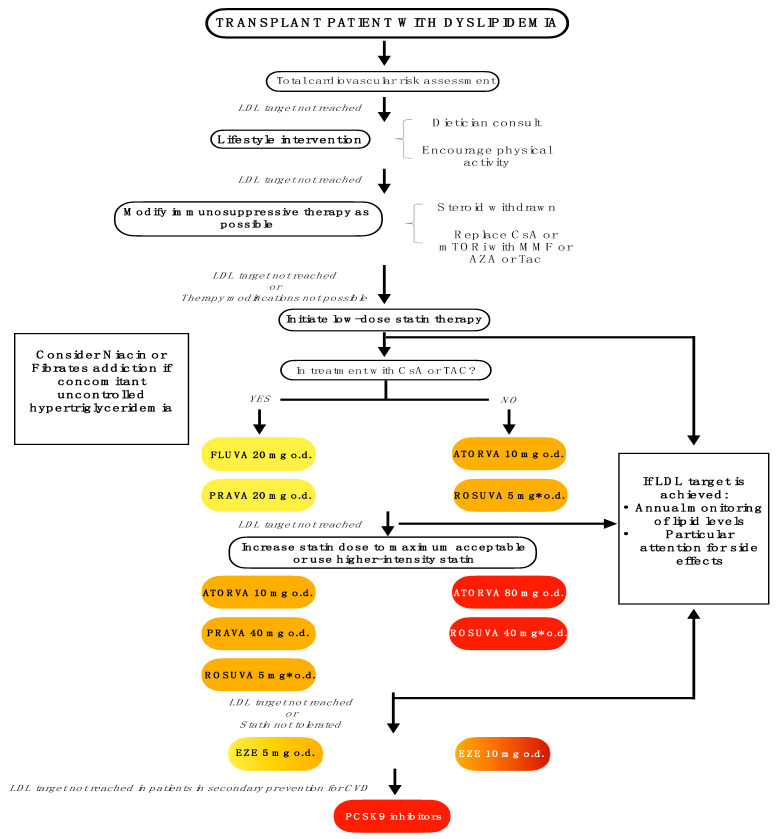
A suggest algorithm to managing dyslipidemia in transplant patients. Atorva: atorvastatin; AZA: azathioprine; CsA: cyclosporine; CVD: cardiovascular diseases; Eze: ezetimibe; Fluva: fluvastatin; o.d.: once daily; LDL: low-density lipoprotein; MMF: mycophenolate mofetil; mTORi: mammalian target of rapamycin inhibitors; PCSK9: Proprotein convertase subtilisin/kexin 9; Prava: pravastatin; Rosuva: rosuvastatin (* should be avoided severe chronic kidney disease); Tac: tacrolimus. Yellow color: low-intensity statin, expected LDL circulating reduction <30%; Orange color: moderate-intensity statin, expected LDL circulating reduction 30–50%; Red color: high-intensity statin or PCSK9i, expected LDL circulating reduction >50%; Color gradient for Eze indicates differences in LDL circulating reduction if ezetimibe is administered alone or co-administered with statin.

Also, in Figure 2 we proposed some therapeutic options and algorithms in different settings.

## 6. Discussion

Organ transplantation represents in many cases the treatment of choice for suitable patients with end-stage diseases [150,151].

Immunosuppressive therapy (cyclosporine, sirolimus, tacrolimus, etc.,) used in solid organ transplant recipients to prevent transplant rejection induces an increase in serum lipid levels. Hyperlipidemia induces nephrotoxicity, glomerulosclerosis, and chronic interstitial nephritis secondary to oxidative stress and progressive renal damage related to monocyte inflammation and mesangial proliferation induced by increased cytokines. Transplant rejection associated with dyslipidemia is the decrease in the immunosuppressive activity of cyclosporine with increased serum lipids that may eventually lead to immune sensitization. 

Dyslipidemia decreases the availability of intracellular concentration of cyclosporine to inhibit immune activation and leads to chronic loss of allograft. Dyslipidemia related to immunosuppressants leads to the decrease in the effect of the immunosuppressant by reducing availability and leading to the loss of the transplant organ. Dyslipidemia, one of the most well-known cardiovascular risk factors, is very common in patients who have undergone organ transplantation and are on immunosuppressant therapy. Their action on lipid metabolism, incorrect nutrition, body weight, renal function, glucose metabolism, and genetic predisposition increase the risk of cardiovascular diseases (ASCVD), which represent the main cause of long-term mortality in these patients.

The management of the different forms of dyslipidemia in transplant recipients is comparable to that recommended for patients with high or very high cardiovascular risk, although extreme attention is required for possible side effects related to drug interactions. According to the 2019 ESC and EAS guidelines for the treatment of dyslipidemia, statins remain the gold standard for treatment, even in these patients; it should be started with low doses and modified in relation to the response and the appearance of adverse effects (mainly hypertransaminasemia and hyperCPKemia) related to drug interactions, especially for some immunosuppressants, such as cyclosporin. In patient’s intolerant to statins, or in those who do not reach the therapeutic target, despite the use of high-intensity statins at the maximum tolerated dose, the addition of ezetimibe can be evaluated, always with careful monitoring of side effects. 

In conclusion, the transplant recipient patient on immunosuppressive therapy requires, as with a normal dyslipidemic patient, a lipid-lowering treatment precisely because of the effect on the lipid metabolism of these drugs. This treatment must be carefully modulated according to the immunosuppressive drugs used and any pharmacological interactions. However, in this case, even more than in the others, the drug therapy becomes targeted, personalized for each patient according to their other specific therapies, always in compliance with the latest international guidelines.

A therapy that simultaneously manages to reduce the patient’s cardiovascular risk and preserve the transplanted organ.

## 7. Conclusions

Thanks to recent advances in transplantation, life expectancy of transplant recipients has extended significantly. For this reason, cardiovascular diseases, rather than transplant rejection, have become the leading cause of death in these patients.

Several factors are responsible for increasing the incidence of cardiovascular diseases, such as obesity, renal function, glucose metabolism, and genetic predisposition, but dyslipidemia due to the side effects of immunosuppressants is the main cause.

Moreover, an increase in LDL and other lipoproteins has been shown to be related to other transplant complications, such as chronic allograft nephropathy and cardiac allograft vasculopathy.

Although each patient has their own target based on comorbidities, LDL levels < 100 mg/dL in all patients are suggested to prevent these events.

Treatment of lipid alteration is more complicated in transplant patients for several reasons. First, the causes of dyslipidemia are the same drugs that make it possible to extend a patient’s life, so they often cannot be reduced or suspended. Moreover, the usual therapies for dyslipidemia, in particular statins which remain the first choice, must be administered carefully for possible drug interactions.

For this reason, dyslipidemia treatment must be one of the priorities in the management of these patients, tailoring therapy as needed and never underestimating the increase in cardiovascular risk that lipid alterations involve.

## Figures and Tables

**Table 1:** Immunosuppressants effects on cardiovascular risk factors.

Immunosuppressants		CV Risk Factors Exacerbated
Corticosteroids	Prednisone	Hyperglycemia (+++), Arterial hypertension (++), Triglycerides (++), LDL (++).
	Deflazacort	Hyperglycemia (+), Arterial hypertension (++), Triglycerides (+), LDL (+).
Calcineurin Inhibitors	Cyclosporine	Hyperglycemia (+), Arterial hypertension (+++), Triglycerides (++), LDL (+++).
	Tacrolimus	Hyperglycemia (+), Arterial hypertension (+++), Triglycerides (+), LDL (++).
mTOR Inhibitors	Sirolimus	Triglycerides (+++), LDL (+++).
	Everolimus	Triglycerides (+++), LDL (+++).
Antiproliferative Agents	Azathioprine	No significant increase
	Mycophenolate Mofetil	No significant increase

mTOR: mammalian target of rapamycin. (+): mild increase; (++): moderate increase; (+++) severe increase. The magnitude of the effect was arbitrarily estimated by the authors according to the evidence discussed in this review.

**Table 2:** Benefits of lipid-lowering agents and dangerous interactions with immunosuppressants.

	Drugs	Dangerous Interaction	Benefit
Statins	Atorvastatin	Co-administered with CsA or Tac increase statin exposure and risk for myopathy	LDL and Tg reduction CVD mortality reduction Plaque stabilization
	Lovastatin	Co-administered with CsA or Tac increase statin exposure and risk for myopathy	
	Simvastatin	Co-administered with CsA or Tac increase statin exposure and risk for myopathy	
	Rosuvastatin	Co-administered with CsA or Tac increase statin exposure and risk for myopathy	
Fibrates	Gemfibrozil	Can reduce plasma levels of CsA and mTORi. Increased risk for myopathy in coadministration with statins	LDL reduction HDL increase
	Fenofibrate	Can reduce plasma levels of CsA and mTORi. Increased risk for myopathy in coadministration with statins	
	Ezetimibe	Co-administered with CsA can increase ezetimibe and CsA levels and risk for side effects	LDL reduction
	Bile acid sequestrants	Can reduce MMF, CsA, Tac and mTORi levels, their administration should be delayed by 4 h from bile acid sequestrants	LDL reduction
	Lomitapide	Can increase plasma levels of CsA, Tac and mTORi	LDL reduction

CsA: cyclosporine; HDL: high-density lipoprotein; LDL: low-density lipoprotein; MMF: mycophenolate mofetil; mTORi: mammalian target of rapamycin inhibitors; Tac: tarolimus; Tg: triglycerides.

## Data Availability

Data sharing not applicable.
